# Multi-User Joint Detection Using Bi-Directional Deep Neural Network Framework in NOMA-OFDM System

**DOI:** 10.3390/s22186994

**Published:** 2022-09-15

**Authors:** Md Habibur Rahman, Mohammad Abrar Shakil Sejan, Seung-Geun Yoo, Min-A Kim, Young-Hwan You, Hyoung-Kyu Song

**Affiliations:** 1 Department of Information and Communication Engineering, Sejong University, Seoul 05006, Korea; 2 Department of Convergence Engineering for Intelligent Drone, Sejong University, Seoul 05006, Korea; 3 Department of Computer Engineering, Sejong University, Seoul 05006, Korea

**Keywords:** 5G, machine learning, Bi-LSTM, wireless communication, NOMA, CNN

## Abstract

Non-orthogonal multiple access (NOMA) has great potential to implement the fifth-generation (5G) requirements of wireless communication. For a NOMA traditional detection method, successive interference cancellation (SIC) plays a vital role at the receiver side for both uplink and downlink transmission. Due to the complex multipath channel environment and prorogation of error problems, the traditional SIC method has a limited performance. To overcome the limitation of traditional detection methods, the deep-learning method has an advantage for the highly efficient tool. In this paper, a deep neural network which has bi-directional long short-term memory (Bi-LSTM) for multiuser uplink channel estimation (CE) and signal detection of the originally transmitted signal is proposed. Unlike the traditional CE schemes, the proposed Bi-LSTM model can directly recover multiuser transmission signals suffering from channel distortion. In the offline training stage, the Bi-LTSM model is trained using simulation data based on channel statistics. Then, the trained model is used to recover the transmitted symbols in the online deployment stage. In the simulation results, the performance of the proposed model is compared with the convolutional neural network model and traditional CE schemes such as MMSE and LS. It is shown that the proposed method provides feasible improvements in performance in terms of symbol-error rate and signal-to-noise ratio, making it suitable for 5G wireless communication and beyond.

## 1. Introduction

In the last decade, wireless communication has been revolutionized by the unprecedented growth of consumer demand. First-generation (1G) wireless communication was implemented for frequency-division multiple access (FDMA), and second-generation (2G) wireless communication was developed for time division multiple access (TDMA) or code division multiple access (CDMA) [1]. In addition, third-generation (3G) wireless communication is used for wideband code division multiple access (WCDMA), and orthogonal frequency division multiple access (OFDMA) is used for fourth-generation (4G) and fifth-generation (5G) communication [1]. The reduction in symbol-error rate (SER) and signal-to-noise ratio (SNR) in the wireless communication system is important in order to improve the system performance. 5G mobile communication has implementation constraints such as high data rates, ultra-low latency, and high reliability [2]. 5G connectivity has a vital role in implementing quality service, big data processing chain, and ubiquitous connectivity for Internet of Things (IoT) devices. Non-orthogonal multiple access (NOMA) is considered a spectral efficient multiple access technique used to enable 5G technology [3]. NOMA has the following capabilities: high spectrum efficiency, low latency, and high connection density for transmitting signals to different users (UEs) on the same frequency and time slot. NOMA-based communication can be broadly classified as power domain NOMA and code domain NOMA [4]. Power domain NOMA achieves multiplexing by allocating different power to the UEs that are in the coverage area. In the case of code domain NOMA, multiplexing occurs in the UEs by employing specific spreading sequences for each user (UE) which has sparse, low-density, and low inter-correlation properties [5]. NOMA-based communication supports multiuser communication in the power domain. By applying superposition coding at the transmitter and successive interference cancelation (SIC) at the receiver, multiple UE data can be extracted on the same communication channel [6,7,8].

Machine learning (ML) techniques can provide significant advantages in performance enhancement in wireless communication [9]. ML can provide solutions to complex problems without explicit programming and the obtained results can be implemented with high accuracy [10]. ML algorithms are provided with example data, and the model can give a prediction after analyzing the data [11]. To take advantage of the ML method, many works have been conducted to solve the issues connected with channel state information (CSI), channel estimation (CE), and signal detection. In this paper, a deep neural network (DNN) which has a bi-directional long short-term memory (Bi-LSTM) based multiuser joint CE and signal detection for NOMA-orthogonal frequency division multiplexing (OFDM) system is proposed.

The contributions of the proposed study can be summarized as follows:

- Multiuser uplink CE and signal detection for NOMA-OFDM wireless communication is considered. The CE and signal detection is performed by the proposed Bi-LSTM model over the Rayleigh fading channel.
- The proposed Bi-LSTM model can jointly estimate and detect the transmission data from multiple UE signals directly instead of the traditional SIC method.
- To observe the effectiveness of the proposed model, a comparative analysis of convolutional neural network (CNN) and the proposed model in terms of SER is performed.
- Using the Monte Carlo simulation, the SER performance of Bi-LSTM is evaluated in terms of different SNR. It is observed that the performance of the proposed model is comparable to the outage performance of the conventional NOMA-SIC methods, including least square (LS) and minimum mean square error (MMSE) and CNN model.

The rest of the paper is organized as follows. Section 3 describes the system model, including the channel model and problem statement. In Section 4, the proposed deep-learning model is described. The results and discussion are presented in Section 5, and the conclusions are drawn in Section 6.

## 2. Related Works

ML has also been used in NOMA communication in the existing literature. The authors of [12] proposed a pilot-assisted learnable SIC model for enhancing bit-error rate performance. Single-input multiple outputs are considered to train the deep-learning network to learn SIC detection parameters. In [13], the authors proposed a DNN based receiver which solves CE error, time delay, and flexible detection of different UE data. The proposed DNN network consists of fully connected layers, a sigmoid function, and a Relu activation function. A CNN-based SIC scheme was proposed in [14]. The results showed that sum rate loss caused by imperfect SIC can be mitigated by the proposed CNN model. The authors of [15] proposed a deep-learning approach for analyzing CSI and detecting the original transmit sequence. The proposed model was built using seven layers of one input layer, one output layer, and five hidden layers. The complex received signal is used as input data and it is mapped to an output group of four alternatives. The study [16] proposed a deep-learning-based receiver for uplink multiple input single output using NOMA. In [17], the authors proposed deep-learning-based method for signal detection and power allocation in NOMA-based futuristic cognitive radio networks. The proposed system was optimized and determined the desired solution in one step without CE via the DNN model. For detection of the modulation order of interference signal in the NOMA system, a ML-based blind detection method was proposed in [18]. In [19], the authors proposed a deep-learning-based UE selection method for a cooperative NOMA system with imperfect CSI. To maximize the sum rate and efficiency of energy, a deep-learning-based multiple input multiple output (MIMO)-NOMA system was proposed in [20]. The proposed model was built with effective communication DNN, which consisted of several convolutional layers and multiple hidden layers. In [21], the authors proposed a DNN based MIMO-NOMA which optimized both precoding and SIC decoding in the sense of minimizing the entire mean square loss of the UEs signal. The SIC method has limitations in the complex multipath channel environment, as well as imperfect interference cancelation and propagation errors. To overcome these problems, a DNN which has a Bi-LSTM for joint CE and signal detection of multiuser NOMA-OFDM is proposed.

## 3. System Model

**Figure 1:**
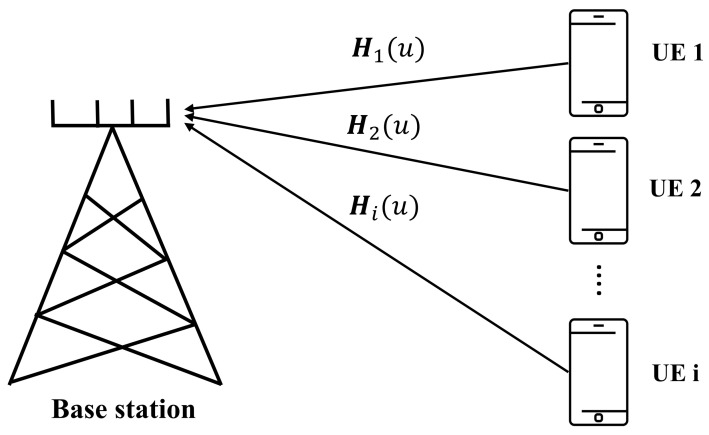
Uplink multiuser communication system with BS.

Figure 1 shows the multiuser uplink communication system with a base station (BS). In this section, first, the channel model and problem illustration are presented. Then, the proposed DNN is described.

### 3.1. Signal and Channel Model

**Figure 2:**
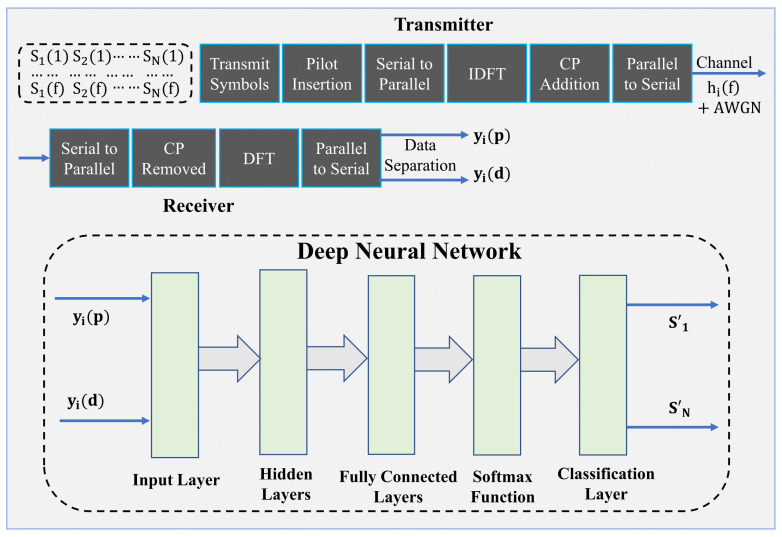
The overview of the proposed DNN-based NOMA-OFDM estimation system.

An uplink multiuser NOMA-OFDM system is considered, where the system consists of a BS and $UE_{i}$ where i=1,2,…,N. At the transmitter, a conventional OFDM-NOMA is used. The BS receives a superposition of symbols from multiple UE with additional channel noise. In the OFDM-NOMA system, a pilot signal is inserted to use CE and signal detection in favor of OFDM. After transmission and reception of data, a DNN model is trained and tested for channel estimation and signal detection. The overview of the proposed system is shown in Figure 2. The superposition coding symbol $Y_{r}$ for *N* UE can be written as follows [15]:

(1) $$Y_{r}=\sum_{i=1}^{N}{\sqrt{\omega }}_{i}s_{i},$$

where $\omega_{i}$ is the power allocation coefficient of $UE_{i}$ and $s_{i}$ is the baseband modulated symbols for $UE_{i}$.

In the general OFDM system, before pilot signal insertion, at the beginning, superposition coding is converted into serial to parallel, and after that, it transformed through inverse discrete Fourier transform (IDFT) [22]. Therefore, the symbol of OFDM-NOMA can be expressed as follows:

(2) $$s_{i}\left(n\right)=IDFT\left\{Y_{r}\left(f\right)\right\}=\sum_{f=1}^{N_{g}}Y_{r}\left(f\right)e^{j(2\pi fn/N_{g})},n=1,2,\ldots ,N_{g},$$

where $N_{g}$ is the number of subcarriers in the frequency domain. In addition, the symbol in the fth subcarrier of OFDM is $Y_{r}\left(f\right)$. To cancel the inter-carrier interference (ICI) among OFDM subcarriers, a cyclic prefix (CP) or guard interval is added to this signal. After adding CP, the OFDM symbol can be expressed as follows:

(3) $$\left\{ \begin{array}{ll} s_{cp}\left(n\right)=s_{i}\left(N_{g}+n\right),n=-N_{cp},-N_{cp}+1,\ldots ,-1 & \\ s_{i}\left(n\right),n=1,2,\ldots ,N_{g}, \end{array}\right.$$

where CP data length is $N_{cp}$. After adding CP to OFDM, it is converted into serial form and is transmitted over the Rayleigh fading channel. However, the received signal at the terminal side is expressed as follows:

(4) $$y_{cpi}\left(n\right)=\sqrt{P}\omega_{i}*h_{i}\left(n\right)s_{cpi}\left(n\right)+G\left(n\right),i=1,2,\ldots ,N,$$

where the transmitted power of $UE_{i}$ is *P*, $h_{i}\left(n\right)$ is the discrete Fourier transform of the impulse response of a multipath channel, and ∗ denotes the convolution operation. The transmitted symbol with CP data for $UE_{i}$ is represented by $s_{cpi}\left(n\right)$. The additive white Gaussian noise (AWGN) G(n) at the receiver is represented as $\mathrm{CN}(0,\sigma^{2})$. The G(n) can be expressed as follows [23]:

(5) $$\begin{array}{c} G\left(n\right)=\frac{1}{\sigma \sqrt{2\pi }}e^{-\frac{{\left(n-\mu \right)}^{2}}{2\sigma^{2}}}, \end{array}$$

where σ is the noise standard deviation and μ is the mean value of the distribution.

After the CP data is removed, the received signal can be written as follows:

(6) $$y_{i}\left(n\right)=y_{cpi}\left(n+N_{cp}\right),n=1,2,\ldots ,N_{g}.$$

The received signal is transformed by discrete Fourier transform (DFT), and it can be expressed as follows:

(7) $$Y_{i}\left(f\right)=DFT\left\{y_{i}\left(n\right)\right\}=1/N_{g}\sum_{n=1}^{N_{g}}y\left(n\right)e^{j(2\pi fn/Ng)},f=1,2,\ldots ,N_{g}.$$

Therefore, in the OFDM system with *N* UEs per subcarrier, the received signal on subcarrier *f* can be written as follows:

(8) $$Y_{i}\left(f\right)=\sum_{f=1}^{N}\sqrt{P}\omega {}_{i}\left(f\right)*h_{i}\left(f\right)s_{i}\left(f\right)+G\left(f\right).$$

The total power allocation coefficient is summed up to one and can be formulated as follows [24]:

(9) $$\sum_{i=1}^{N}\omega_{i}\left(f\right)=1.$$

The scalar $h_{i}\left(f\right)$ DFT of the impulse response of multipath channel $H_{i}\left(t\right)$ for the $UE_{i}$ can be expressed as follows [25]:

(10) $$H_{i}\left(t\right)=\sum_{r=1}^{R}\upsilon_{i,r}\eta \left(t-\tau_{i,r}\right),$$

where the complex channel gain and corresponding time delay for the *r*th multipath parameters of the $UE_{i}$ are represented by $\upsilon_{i,r}$ and $\tau_{i,r}$, respectively. In this proposed paper, the total number of resolved paths *R* is considered to be 20, and the channel is modeled by the Rayleigh fading.

The traditional SIC methods such as LS and MMSE are used, and are also applied for CSI estimation and detection of the signal [26]. In advance, the correction coefficient $R_{hh}$ for MMSE estimation is calculated. The traditional MMSE estimator can be expressed as follows:

(11) $${\hat{h}}_{\mathrm{MMSEi}}=R_{hY}R_{YY}^{-1}Y=R_{h{\hat{h}}_{\mathrm{LSi}}}{\left(R_{hh}+\frac{\sigma_{w}^{2}}{\sigma_{x}^{2}}I\right)}^{-1}{\hat{h}}_{\mathrm{LSi}},\;\;i=1,2,$$

where the MMSE estimated channel from the *i*th transmit antenna is ${\hat{h}}_{\mathrm{MMSEi}}$, $Rhh=E\{hh^{H}\}$ is the autocorrelation matrix, the cross correlation between the true channel and estimated channel by LS estimation is represented $R_{h{\hat{h}}_{\mathrm{LSi}}}=E\left\{h{\hat{h}}_{LSi}^{H}\right\}$, the transmitted signal variance is $\sigma^{2}$, and the identity matrix is *I*. In addition, since more power is allocated to the $UE_{i}$ signal, a ML detector is used to predict signals [27]. In addition, based on the UE CSI, SIC is implemented. Every UE sends pilots symbol to the BS, and these pilots are utilized for the CE and SNR inference [6,28]. After estimation of the first UE signal, the second UE signal $Y_{2}^{\prime }\left(f\right)$ can be estimated as follows:

(12) $$Y_{2}^{\prime }\left(f\right)=Y\left(f\right)-\sqrt{P}\omega {}_{1}\left(f\right){\hat{h}}_{1}\left(f\right){\hat{s}}_{1}\left(f\right).$$

### 3.2. Problem Illustration

To estimate and detect the multiuser NOMA signal, a Bi-LSTM model is proposed. The different UEs signals interfere with each other conventionally. Using the SIC technique, the cancelation of the stronger signal is imperfect. To address this limitation, joint detection is proposed in order to learn the Rayleigh channel and multiuser signal detection. The Rayleigh fading channel $H_{i}\left(t\right)$ for the multipath environment can be expressed as follows:

(13) $$\begin{array}{c} H_{i}\left(t\right)=\sum_{r=1}^{R}e^{j(2\pi f_{a}t+\phi_{a})}\upsilon_{i,r}\eta \left(t-\tau_{i,r}\right), \end{array}$$

where $f_{a}$ is the Doppler frequency (DF) shift and $\phi_{a}$ is the DF phase of the *r*th path, respectively. The mathematical expression of DF shift can be written as $f_{a}=\left(v/c\right)f_{c}sin\theta_{a}$, where *v* is the speed of UE, *c* is the light speed, $f_{c}$ is the carrier frequency, and the angle between UE and incident signal is represented by $\theta_{a}$. The transmitted symbols are considered the true values, and the received signals are treated as the input to the training model.

## 4. Proposed Deep-Learning Model

In this section, the proposed Bi-LSTM model input data preparation and model structure with its operation in the NOMA OFDM framework are discussed. Then, the offline training and online testing mechanism of the trained model are presented.

### 4.1. Data Generation

In this paper, the OFDM with 64 subcarriers is considered. Each OFDM packet consists of two pilots and one data symbol. The quadrature phase shift-keying (QPSK) modulation is considered, and each symbol consists of 2 bits per subcarrier. After the IDFT is performed and CP data is added as a guard interval to avoid inter-symbol interference, the OFDM packet is transmitted through the Rayleigh channel. The BS receives the sum of the OFDM packet from the multiuser with noise.

The received OFDM packet is stored as the training data sample by creating a feature vector $y_{u}$. The feature vector $y_{u}$ is constructed with the real and imaginary values of all the symbols in the OFDM packet. The total training sample is comprised of the multiplication of the number of total data packets and the number of labels. The model can be trained to restore data on an arbitrary subcarrier *f* by using the corresponding B(f) in the training. The system has a total number of $2^{4}$ combinations or labels for UEs transmitting QPSK symbols. The total label can be expressed as $B\left(f\right)=1,2,3,4,\ldots ,N_{l}$ for $N_{l}=16$. As the 64 subcarriers are considered, one OFDM packet contains 3 OFDM symbols and two active UEs. The input size of the training model is 64×3×2=384. In total, 50,000 data packets are used and the total data samples are comprised of 50,000 × 16 = 800,000 which are generated to train the model. The total generated data sample is split into 2 sizes, such as train data size and validation data size, to justify the efficiency of the model. The sizes of training and validation data samples are (4/5), i.e, 640,000 and (1/5), i.e, 160,000, respectively.

### 4.2. Model Architecture

#### 4.2.1. Network Description

**Figure 3:**
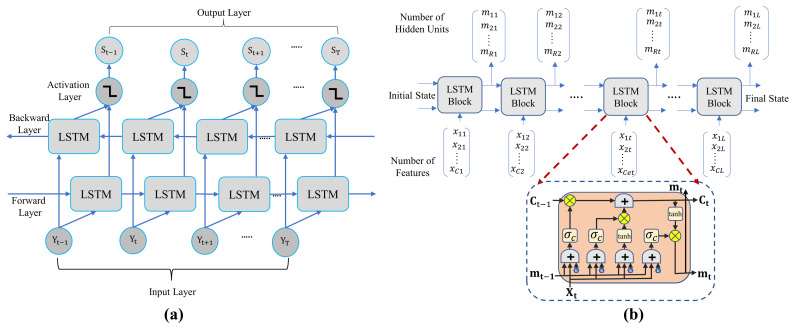
(**a**) The architecture of the Bi-LSTM model system with its different layers. (**b**) The internal cell structure of the LSTM model.

The Bi-LSTM is comprised of the forward and backward directions of the LSTM network [29,30]. It can use information from both sides because the input flows in both directions, as shown in Figure 3a. The forward and backward layers are comprised of two cyclic neural networks which can connect the output layers simultaneously. The output is able to acquire the before and after sequence information of every point. In addition, it explores the relationship between them through training. The accuracy of CE can be improved by this operation. To achieve the CE and signal detection, directional LSTM, which is a special kind of recurrent neural network that consists of the cascade of LSTM cells, is exploited [31]. The LSTM network comprises 4 layers, including LSTM hidden layers, fully connected layers, softmax function layers, and classification layers. The LSTM hidden layer is implemented with 100 hidden units. In the LSTM hidden layers, the learnable weights includes input weights *w*. The recurrent weights are *T*, and *b* is the bias. The second layer is a fully connected layer that contains a 16 number of classes. The fully connected layer is implemented to sequence and time-series data for classification. The output of the LSTM layers is processed by the fully connected layer. A fully connected layer adds a bias vector *b* to the input after multiplying it by a weight matrix *w*. Thus, it estimates all components of the complex modulated signal of each UE. All neurons in the fully connected layer are connected to all neurons in the previous layer. This brings together all the properties and information collected from the previous layer. The fully connected layer works individually on every time step in the LSTM network. To derive the outputs for the terminal layer, the softmax activation function is used. In the last layer, the classification layer is utilized to map the output to a vector probability and a fully connected layer with an output size equal to the number of classes is specified, and then the error between them is passed as feedback for the training. Finally, the mean-squared error (MSE) for the overall network to detect at $UE_{i}$ is expressed as follows [32]:

(14) $$MSE=\frac{1}{Q}\sum_{q=1}^{Q}{\left(S_{i}\left(q\right)-{\hat{S}}_{i}\left(q\right)\right)}^{2},$$

where the number of training OFDM samples is represented by *Q*, $S_{i}\left(q\right)$ is the target output, and ${\hat{S}}_{i}\left(q\right)$ is the predicted output at the response *q*. As a means of minimizing the loss, the well-known Adam optimization algorithm is utilized [33].

#### 4.2.2. Internal Structure of LSTM

The LSTM network can learn information between time steps of sequence data and preserve relevant information. The time steps are treated equivalently as subcarriers in the OFDM system. By focusing on one time-step module in the LSTM layer, the DNN can be trained to realize multiuser detection for an arbitrary subcarrier. The internal cell structure and operation of LSTM network are shown in Figure 3b [34]. The output of the LSTM cell is generated according to the current input and the preceding cell state. The LSTM cell consists of three gates, such as the forget gate, the input gate, and the output gate. In addition, the LSTM is comprised of two states, namely cell state $C_{t-1}$ and hidden state $m_{t-1}$. The cell state works as a memory to cumulate information that is extracted from past inputs. On the other hand, to compute the output, the hidden state is utilized. From the Figure 3b, *t* is the time instant, the current input is $x_{t}$, and finally, the current output channel coefficient of multiuser at time *t* is denoted by $m_{t}$. The LSTM cell can add and remove information from the cell state at each time step, which is updated through operation of the gates. The operations of each gate can be summarized as follows:

The control for the level of cell state that needs to be reset is performed by the forget gate. The forget gate $fr_{t}$ can be expressed as follows [35]:

(15) $$fr_{t}=f_{\sigma_{c}}\left(w_{fr}x_{t}+T_{fr}m_{t-1}+b_{fr}\right),$$

where $w_{fr}$ is the weight related to $x_{t}$ and $T_{fr}$ is the weight related to $m_{t-1}$. The bias of the forget gate is $b_{fr}$. The control for the level of cell state that needs to be updated is performed by the input gate. The input gate $in_{t}$ can be expressed as follows [35]:

(16) $$in_{t}=f_{\sigma_{c}}\left(w_{in}x_{t}+T_{in}m_{t-1}+b_{in}\right),$$

where $w_{in}$ is the weight related to $x_{t}$ and $T_{in}$ is the weight related to $m_{t-1}$. The bias of the input gate is $b_{in}$. The addition of information to the cell state is managed by the candidate gate. The candidate gate $ca_{t}$ can be expressed as follows [35]:

(17) $$ca_{t}=f_{\tanh}\left(w_{ca}x_{t}+T_{ca}m_{t-1}+b_{ca}\right),$$

where $w_{ca}$ is the weight associated with $x_{t}$ and $T_{ca}$ is the weight related to $m_{t-1}$. The bias of the candidate gate is $b_{ca}$. The updated cell state can be expressed as follows:

(18) $$up_{t}=\left(C_{t-1}⊙fr_{t}\right)+\left(in_{t}⊙ca_{t}\right),$$

where the element-wise multiplication is represented by ⊙. The control for the level of cell state to be updated is performed by the output gate. The output gate $ou_{t}$ can be expressed as follows:

(19) $$ou_{t}=f_{\sigma_{c}}\left(w_{ou}x_{t}+T_{ou}m_{t-1}+b_{ou}\right),$$

where $w_{ou}$ is the weight related to $x_{t}$ and $T_{ou}$ is the weight associated with $m_{t-1}$. The bias of the output gate is $b_{ou}$. $f_{\sigma_{c}}\left(z\right)=\left[1/\left(1+e^{{}_{z}}\right)\right]$ is the sigmoid function, which is responsible for computing the gate activation function. The estimated output coefficient of the hidden state at time step *t* can be expressed as follows:

(20) $$m_{t}=ou_{t}⊙f_{\tanh}\left(up_{t}\right),$$

where $f_{\tanh}\left(z\right)=\left[\left(e^{2z}-1\right)/e^{2z}+1\right)]$ is the hyperbolic tangent function, which is responsible for computing the state activation function.

Figure 3a shows the Bi-LSTM model, which is constructed with two LSTM layers in opposite directions. The output $V_{t}$ of the two hidden Bi-LSTM state layers can be calculated as follows [36]:

(21) $$V_{t}=f\left(w_{V\vec{T}}{\vec{T}}_{t}+w_{V\overset{\leftarrow }{T}}{\overset{\leftarrow }{T}}_{t}+b_{Z}\right),$$

where $\vec{T}$ and $\overset{\leftarrow }{T}$ are the forward and backward sequences.

#### 4.2.3. Offline Training and Online Testing Operation of the Model

**Figure 4:**
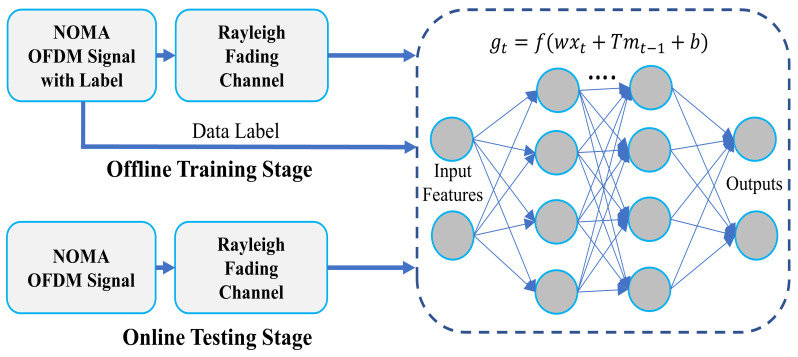
Training and testing process of the proposed model.

**Figure 5:**
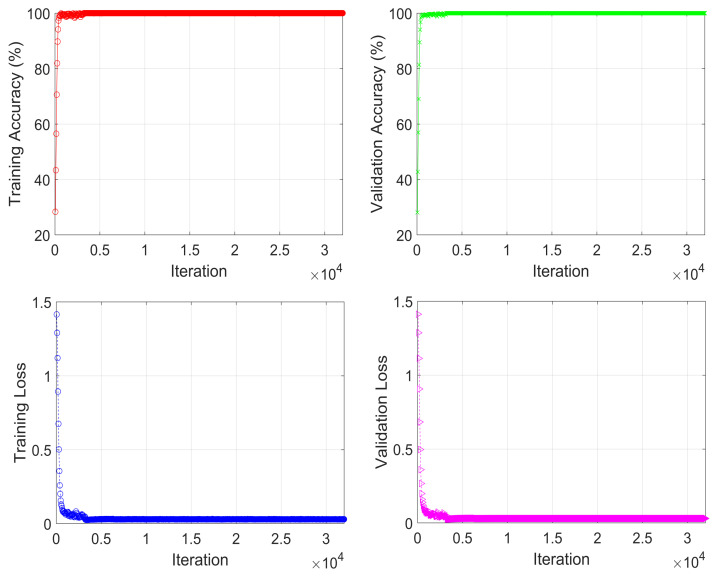
Training and validation progress of the proposed model.

Based on the generated data and the proposed model, the training process is carried out in the offline stage, as shown in Figure 4. The input of the model training system is combined with the received NOMA-OFDM signal as an input layer, and corresponding labels are used as supervised data to assist the DNN in optimizing the settings. The training process of the proposed model is summarized in Algorithm 1. Table 1 shows the training and optimized parameters. Results for training and validation accuracy versus its loss progress during learning of the model are illustrated in Figure 5, where the validation accuracy is 99.90% with the setting of training parameters of minibatch size 2000, epoch 100, and learning rate 0.01.
**Algorithm 1** BiLTSM Training Process1:Load the training and validation data samples.2:Initialize model parameters such as minibatch size, maximum epochs, learning rate.3:Train the model network accordingly and calculate the accuracy error by (14).4:Adam optimization algorithm is used to compute the corrective parameter and to search for the optimal solution with update of the parameters.5:Result: Trained model.6:Save the model.

#### 4.2.4. Testing Process

The online testing process is performed after successive training of the proposed model. The testing process of the proposed model using test datasets is shown in Figure 4. The outage performance and simulation results of the proposed model are evaluated and described in the Section 5.1.

## 5. Simulation Results and Discussion

In this section, the simulation results for the proposed Bi-LSTM model of multiuser CE and signal detection in the NOMA-OFDM system are discussed.

### 5.1. Performance Evaluation

In this section, the performance of the proposed model in the NOMA-OFDM is presented. The simulation work of the proposed Bi-LSTM model based multiuser CE and signal detection is performed using the simulation parameters, as shown in Table 1. The data generation for training the model is discussed in the previous Section 4.1. The SNR value of 30 dB is set up during the generation of training datasets. To generate the datasets and test, the learned model, the CP size 20, and the pilot symbols 64 in each transmitted package are considered. The comparison of CNN, ML, traditional MMSE, and LS methods with the proposed model are performed according to SER versus SNR. In the online testing stage, the [0: 2: 30] dB SNR range is considered for the simulation performance evaluation. Monte Carlo simulation is performed for the evaluation of the SER performance of the proposed Bi-LSTM model.

### 5.2. Simulations Results

**Figure 6:**
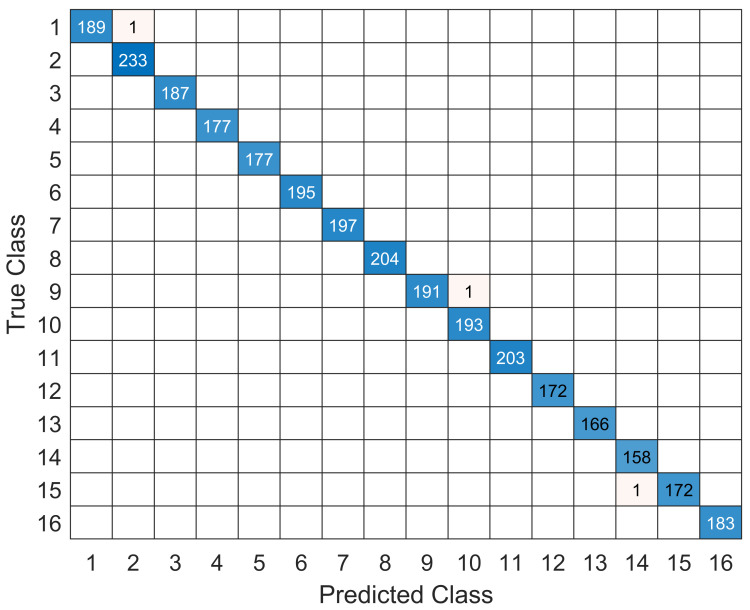
The confusion matrix of the true and predicated class of the model.

To conduct the simulation performance, the proposed model is compared with different traditional CE and signal detection models and a one-dimensional CNN model. A confusion matrix simulation result is performed to observe the symbol classification robustness of the proposed model. Figure 6 shows the confusion matrix for symbol classification according to the number of labels during the testing process of the trained model. The symbol decoding and classification rate of the proposed model are very high in order of true class and predicted class, except for some minor missing classes.

**Figure 7:**
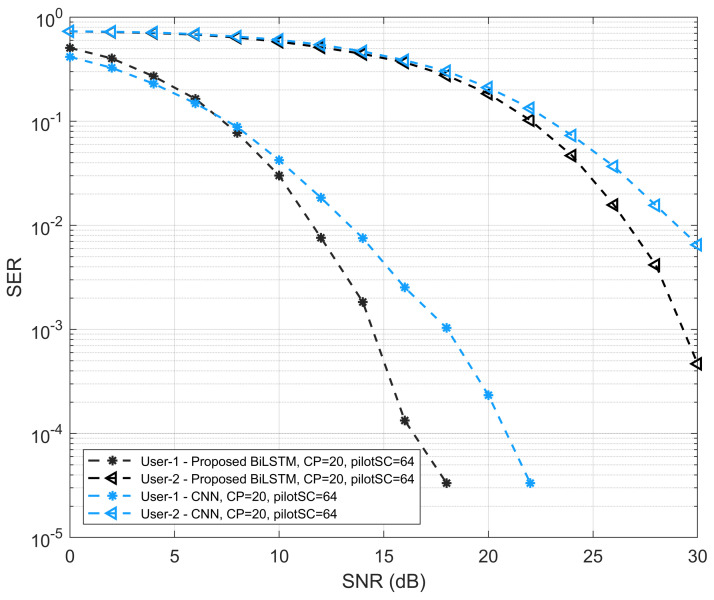
SER versus SNR performance of proposed Bi-LSTM model and CNN model.

Figure 7 shows the simulation results of SER performances for $UE_{1}$ and $UE_{2}$. The output is taken by comparing the proposed network and the CNN model. The comparison of the proposed model and CNN is performed with the same simulation parameters. It can be seen that the SER performance of the CNN model is lower than the proposed Bi-LSTM model. The proposed detection network outperforms the CNN for both UE cases. In addition, with an increase in the SNR values, it can be seen that the SER performance is improved.

**Figure 8:**
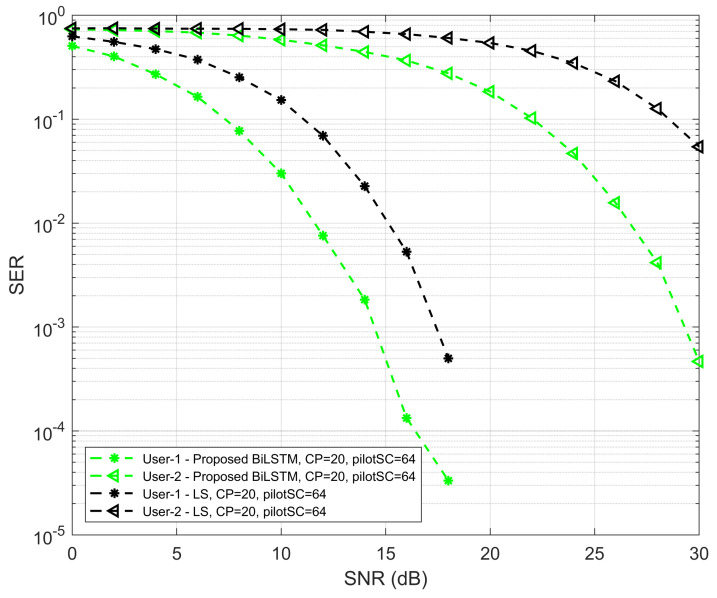
SER versus SNR performance of proposed Bi-LSTM model and LS method.

**Figure 9:**
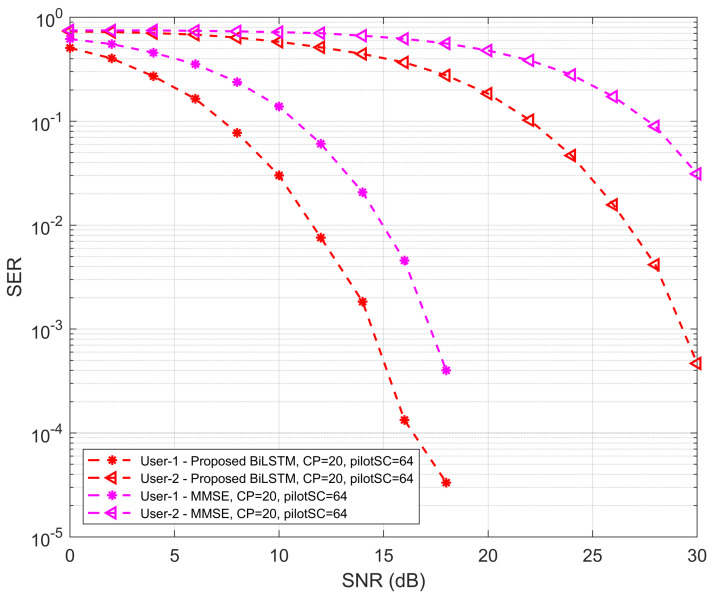
SER versus SNR performance of proposed Bi-LSTM model and MMSE method.

In Figure 8, the SER performances of the proposed network with the traditional LS method for $UE_{1}$ and $UE_{2}$ are well investigated. The comparison of the proposed model and conventional LS is performed using the SER and SNR curve. It is evident that the SER performance of the traditional methods is lower than the proposed BiLTSM model for both UEs with respect to the SNR values. The SER performance of the proposed Bi-LSTM model with traditional MMSE is shown in Figure 9. It is also shown that the SER performance of the proposed Bi-LSTM model is always higher than the MMSE method for both UEs.

**Figure 10:**
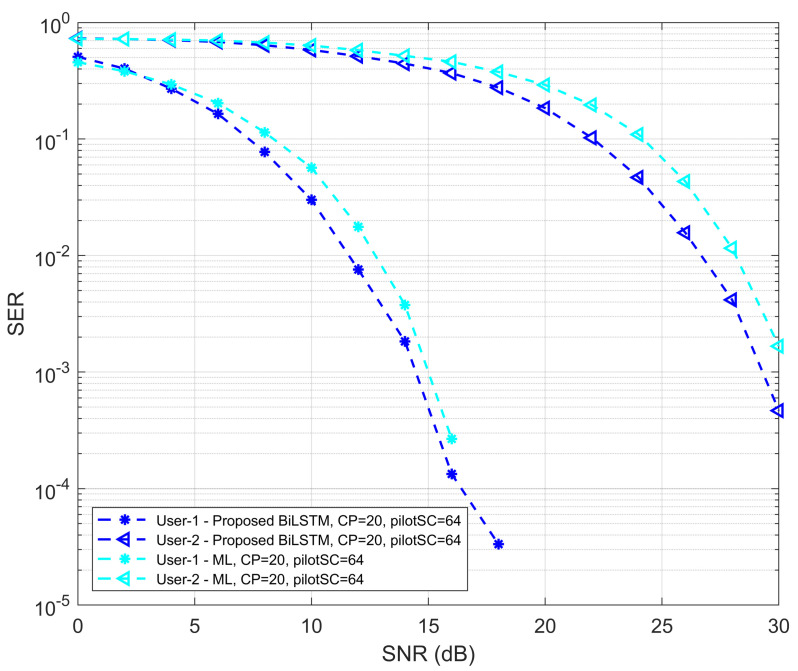
SER versus SNR performance of proposed Bi-LSTM model and ML method.

**Figure 11:**
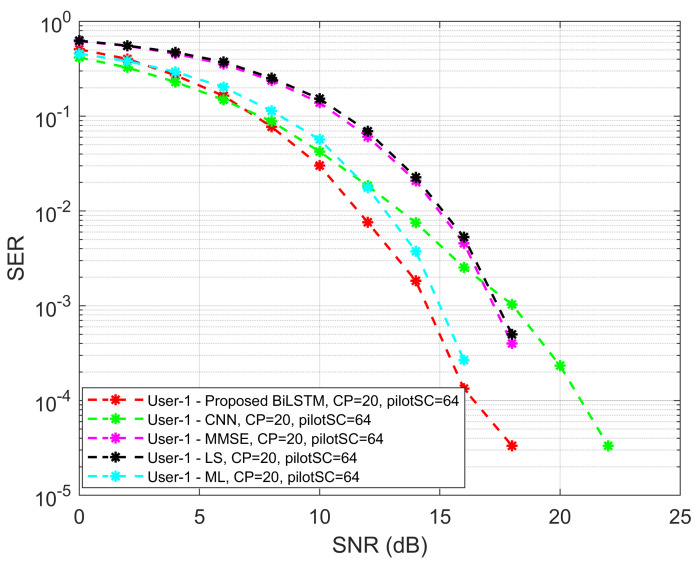
SER versus SNR performance comparison for UE 1 of the proposed method against CNN, LS, MMSE, and ML.

**Figure 12:**
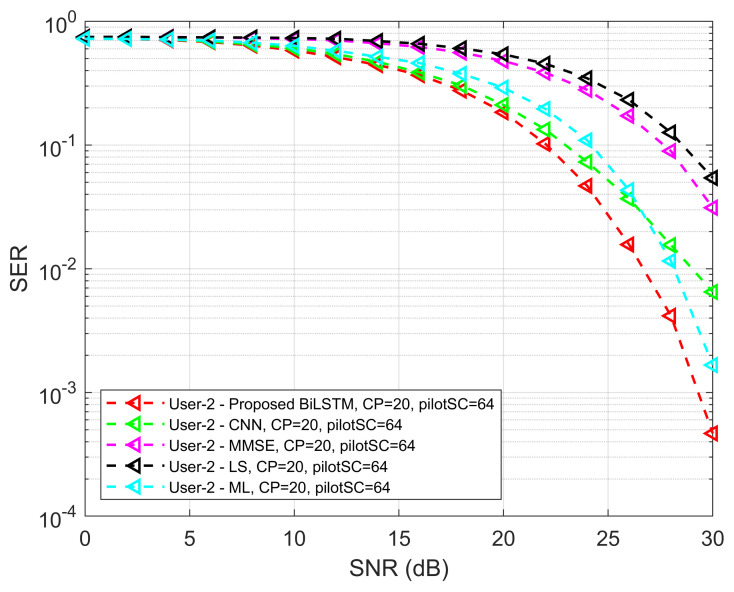
SER versus SNR performance comparison for UE 2 of the proposed method against CNN, LS, MMSE, and ML.

To investigate SER performance, the proposed model is compared with the ML method. Figure 10 shows the SER performance of the proposed model with the ML method. It is seen that the SER performance of the proposed Bi-LSTM model is improved compared to the ML method for both UEs. For $UE_{1}$, at the beginning of the curve with low SNR values, the performance of the proposed model is slightly degraded. In the overall scenarios, the performance of the ML method is lower than the proposed detection network, except for small degradation, but higher than other MMSE and LS methods. In addition, the performance comparison of the proposed model against CNN, LS, MMSE and ML for $UE_{1}$ and $UE_{2}$ shown in Figure 11 and Figure 12, respectively.

**Figure 13:**
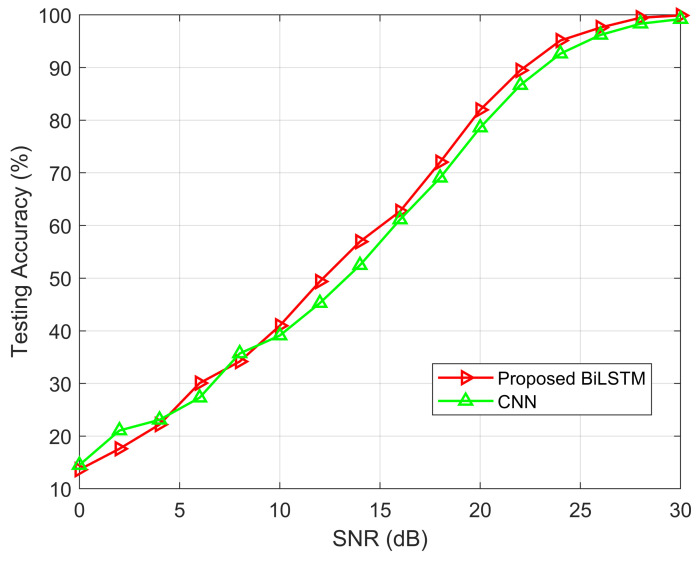
Testing accuracy performance of Bi-LSTM and CNN model.

To observe the learning capability of the proposed model, the simulation results are performed in terms of testing accuracy and SNR (0–20) dB ranges of the last iteration of Monte Carlo simulations. The testing accuracy of the proposed Bi-LSTM and CNN model with different SNR values are shown in Figure 13. It is seen that the testing accuracy has a small amount of variation at the beginning of SNR. After that, the proposed Bi-LSTM model testing accuracy outperforms the CNN model during the measurement of the SER performance with SNR values. It is shown that the proposed model has the robustness of inference capability.

### 5.3. Complexity Analysis

In this section, the computational complexity of the proposed model is described. The computational complexity time is measured by considering the number of floating-point operations (FLOPS). In the proposed model, the Bi-LSTM network has four gates that need to be processed at the same time. The complexity of the detection network is the sum of the four gate parameters and considers the forward and backward transfer process. Accordingly, the computational complexity of the proposed network can be expressed as $O(4\times L_{s}\times O_{s}\times 2\left(h_{l}\times h_{d}\times n_{l}\right))$. Here, $L_{s}$ is the number of received input packets, $O_{s}$ is the OFDM block size, $h_{l}$ is the input size of LSTM, $h_{d}$ is the number of hidden layers, and $n_{l}$ is the neuron size in the LSTM layer.

## 6. Conclusions

In this paper, a Bi-LSTM model-based multiuser uplink CE and signal detection for the NOMA-OFDM system are proposed. Compared with the traditional SIC schemes, the proposed model provides better CE and signal detection performance. The conventional CE methods such as MMSE, LS, and ML are less robust than the proposed Bi-LSTM network in terms of signal recovery. Moreover, to observe the SER performance, the proposed model is compared with the CNN model with different SNR values. It is seen from the simulation results that the SER detection performance rate of the proposed system is also extensively high compared with traditional methods and the CNN model. The proposed model receiver is combined with CE, equalization, and demodulation in an end-to-end model. The proposed model is a good solution for 5G wireless communication and beyond. In the future, this system can be applied in a more complex system such as a MIMO-based NOMA system. It can also be applied to a promising physical layer, such as reflecting intelligent surfaces.

## Figures and Tables

**Table 1:** The simulation parameters.

Parameter	Value
Simulation tool	MATLAB Deep-learning toolbox^TM^
Operating system	Windows 10 Pro
OFDM subcarriers	64
Pilot symbols	64
Channel path	20
Noise	AWGN
Length of CP	20
Channel fading	Rayleigh channel
NOMA UEs	2
Modulation type	QPSK
Total number of packets	50,000
Total model layers	5
Epochs number	100
Learning rate	0.01
Minibatch size	2000
Optimizer	ADAM

## Data Availability

Not applicable.
